# Laparoscopic Heller myotomy is not superior to pneumatic dilation in the management of primary achalasia: Conclusions of a systematic review and meta-analysis of randomized controlled trials

**DOI:** 10.1097/MD.0000000000005525

**Published:** 2017-02-17

**Authors:** Ji-Wei Cheng, Yin Li, Wen-Qun Xing, Hong-Wei Lv, Hao-Ran Wang

**Affiliations:** Department of Thoracic Surgery, The Affiliated Cancer Hospital of Zhengzhou University, Henan Cancer Hospital Zhengzhou, Henan, PR China.

**Keywords:** achalasia, laparoscopic Heller myotomy, pneumatic dilation

## Abstract

**Background:**

Achalasia is an esophageal motility disorder, of unknown cause, which results in increased lower esophageal sphincter tone and symptoms of difficulty swallowing. Current major therapeutic options include laparoscopic Heller myotomy (LHM) and pneumatic dilation (PD). We undertake a systematic review comparing the efficacy and safety of these 2 treatments in the treatment of esophageal achalasia.

**Methods:**

PubMed, Embase, and Cochrane Central Register of Controlled Trials were searched for randomized controlled trial investigating LHM versus PD in the treatment of primary achalasia. The primary outcome was symptom remission rates. The Mantel–Haenszel method with fixed-effect or random-effects model was used to calculate relative risks and 95% confidence intervals (CIs).

**Results:**

Five studies involving 498 participants were included. The cumulative remission rate was significantly higher with LHM at 3 months and 1 year (short-term), with a risk ratio of 1.16 (95% CI 1.01–1.35, *P* = 0.04) and 1.14 (95% CI 1.02–1.27, *P* = 0.02), respectively. There were no significant differences between LHM and PD in 2-year and 5-year remission rate (long-term), with a risk ratio of 1.05 (95% CI 0.91–1.22, *P* = 0.49) and 1.17 (95% CI 0.84–1.64, *P* = 0.34), respectively. Rates of major mucosal tears requiring subsequent intervention with LHM were significantly lower than those of esophageal perforation with PD requiring postprocedural medical or surgical therapy, with a risk ratio of 0.25 (95% CI 0.08–0.81, *P* = 0.02). Postprocedural rates of gastroesophageal reflux, lower esophageal sphincter pressures, and quality of life scores did not differ in trials with sufficient data.

**Conclusions:**

There were no significant differences between LHM and PD in 2-year and 5-year remission rate. This study indicates that either treatment can be proposed as initial treatment for achalasia.

## Abstract 2

Supplemental Digital Content is available in the text

## 1 Introduction

Achalasia is an esophageal motility disorder of unknown cause that manifests as symptoms of difficulty swallowing (dysphagia), with stasis of food and secretions in the lower esophagus. The condition is characterized by the obvious degeneration of inhibitory neurons in the myenteric plexus of the lower esophageal sphincter (LOS). This leads to loss of peristalsis in the distal esophagus and a lack of coordinated LOS relaxation in response to swallowing; finally, the basal tone of the sphincter increases.^[1]^ The annual incidence has been estimated at approximately 1:100,000.^[2,3]^ The most common symptoms include dysphagia, regurgitation, and chest pain; onset of symptoms is often insidious, usually between the ages of 25 and 60 years, and symptoms gradually progress over a period of years.^[4]^ The diagnosis of achalasia may be suspected from the clinical history, confirmed by radiographic, endoscopic, and manometric assessment.^[5]^ The degenerated myenteric plexus neurons cannot restore their function; therefore, reducing the tone of the LOS is the aim of treatments. These include surgical myotomy, endoscopic pneumatic dilation (PD), intrasphincteric botulinum toxin injection, and pharmacological therapy. Pharmacological treatment is reserved for patients with mild symptoms or who refuse other treatments that have little effect.^[6]^ Intrasphincteric botulinum toxin has been shown to be inferior compared to PD at relieving symptoms, and to be less durable.^[7]^ Currently, treatment consists mainly of laparoscopic Heller myotomy (LHM) and PD.

Previous reviews and meta-analyses have suggested that surgical myotomy is the most effective therapy.^[6,8,9]^ However, recent evidence from a large, prospective, multicenter, randomized controlled study comparing LHM with PD has challenged this view by demonstrating equivalent results for both treatments at 2 and 5 years.^[10,11]^ The purpose of this meta-analysis is to compare the efficacy and safety of 2 treatments for patients with achalasia.

## 2 Methods

All analyses were based on previously published studies; thus, no ethical approval and patient consent are required.

### 2.1 Criteria for considering studies for the present review

#### 2.1.1 Types of studies

The studies included in the present review comprise randomized controlled trials (RCTs), with or without blinding, comparing LHM to endoscopic PD in the treatment of achalasia.

#### 2.1.2 Types of participants

The participants in the present review were individuals of any age diagnosed with achalasia (previously untreated or having undergone only an attempt at pharmacotherapy) by a combination of clinical, endoscopic, radiographic, or manometric investigations.

#### 2.1.3 Types of outcome measures

##### 2.1.3.1 Primary outcomes

The primary outcome was symptom remission rates within 3 months, and 1, 2, and 5 years.

##### 2.1.3.2 Secondary outcomes

The secondary outcomes were as follows:

1. Posttreatment complications directly related to the therapy
2. LOS pressure confirmed by esophageal manometry
3. Rates of development of gastroesophageal reflux (GER)
4. Quality of life postintervention
5. Cost-effectiveness

### 2.2 Search methods for identification of studies

PubMed, Embase, and Cochrane Central Register of Controlled Trials databases were searched for records reporting the effect of LHM versus that of PD in the treatment of primary achalasia. The search strategy is shown in Supplemental Content 1. No language restriction was imposed. Publications from January 1, 1975, to March 16, 2016, were considered for review. Two independent investigators carried out the initial search, deleted duplicate records, screened the titles and abstracts for relevance, and identified the publications as excluded or requiring further assessment. Then we reviewed the full-text articles for inclusion. We also manually checked the references of the retrieved articles and previous reviews to identify additional eligible studies.

### 2.3 Data collection and analysis

Data extraction and quality control were done independently by 2 reviewers; κ scores were measured to assess the agreement between the 2 initial reviewers in each step and interpreted as described elsewhere.^[12]^ Any disagreements were resolved by discussion.

### 2.4 Assessment of risk of bias in included studies

#### 2.4.1.1 Quality assessment of trials

Two review authors independently assessed the methodological quality of the selected trials using the following criteria:

1. The method of randomization
2. Allocation concealment
3. Baseline comparability of study groups
4. Blinding and completeness of follow-up

We did not use blinding of participants or intervention providers as an assessment criterion given the nature of the interventions being studied. Trials were graded as follows: A, adequate; B, unclear; and C, inadequate on each criterion, Thus, each RCT was graded as having low, moderate, or high risk of bias. If it was unclear whether a criterion had been met, we sought further information from the author. Any disagreements were resolved by discussion.

### 2.5 Statistical analyses

Summary outcomes are described as proportions and 95% confidence intervals (CIs) for the categorical and weighted mean difference ± standard deviation for continuous data. Cumulative response rates in each group were calculated separately by using the sum of the responders and the total number of included patients and were reported as proportions and CIs for each individual modality. A meta-analysis of intention-to-treat data was done. *P* values <0.05 were considered significant. The significance and the extent of statistical heterogeneity were calculated by using the Q test and I^2^ index, respectively. Random-effect modeling was applied if the *P* value for the test of heterogeneity was <0.10 by using the DerSimonian and Laird method.^[13]^ Risk ratios were calculated for each analysis with the corresponding 95% CIs. Funnel plots were used to detect the possibility of publication bias by evaluating the asymmetry. We also planned to perform sensitivity analyses based on the quality and weight of the trials by excluding each individual trial in turn.

All statistical analyses were done by using RevMan version 5.3 (The Nordic Cochrane Centre, Copenhagen, Denmark).

## 3 Results

### 3.1 Characteristics of included trials

**Figure 1:**
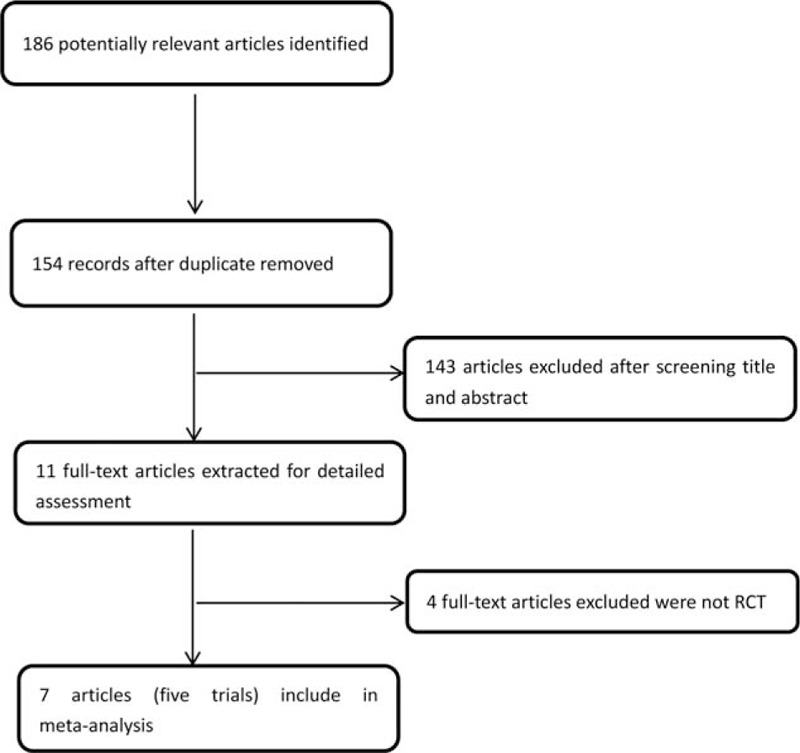
Flowchart of literature search and selection. RCT = randomized controlled trial.

We identified studies using the search criteria and assessed 7 full-text articles for eligibility, as shown in Fig. 1. Four articles actually were from 2 studies^[10,11,14,15]^; therefore, there were 5 studies included in the meta-analysis. Table 1 shows characteristics for each trial. All included studies used graded PD advancing from a 30-mm balloon to a 35-mm balloon and eventually used 40-mm balloons with slightly different criteria. The use of nonvalidated symptom scores presented a limitation of most of the included studies, except the Dakkak score used by Kostic et al^[14]^ and Persson et al,^[15]^ and the Eckardt score used by the European achalasia trial.^[11]^ The duration of follow-up varied from 1 to 78 months.

**Table 1:**
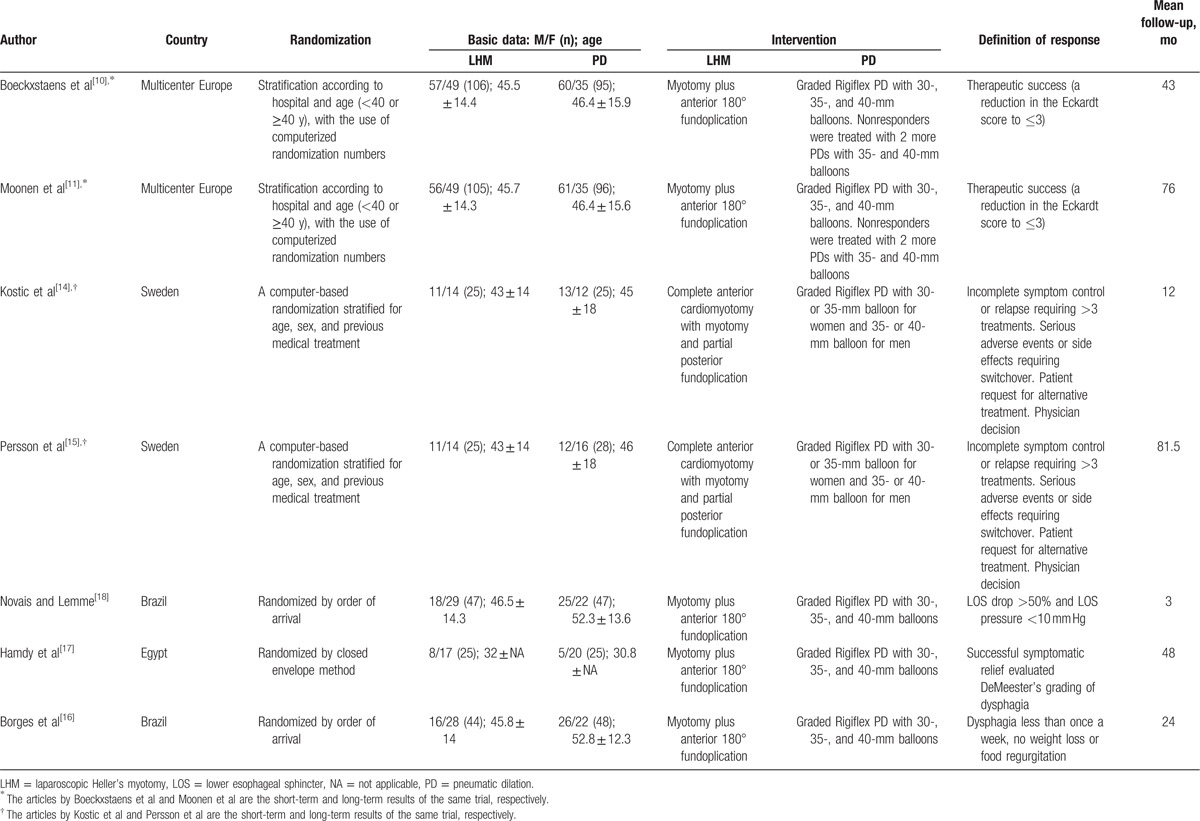
Characteristics of the included studies (n = 7).

### 3.2 Risk of bias in included studies

**Figure 2:**
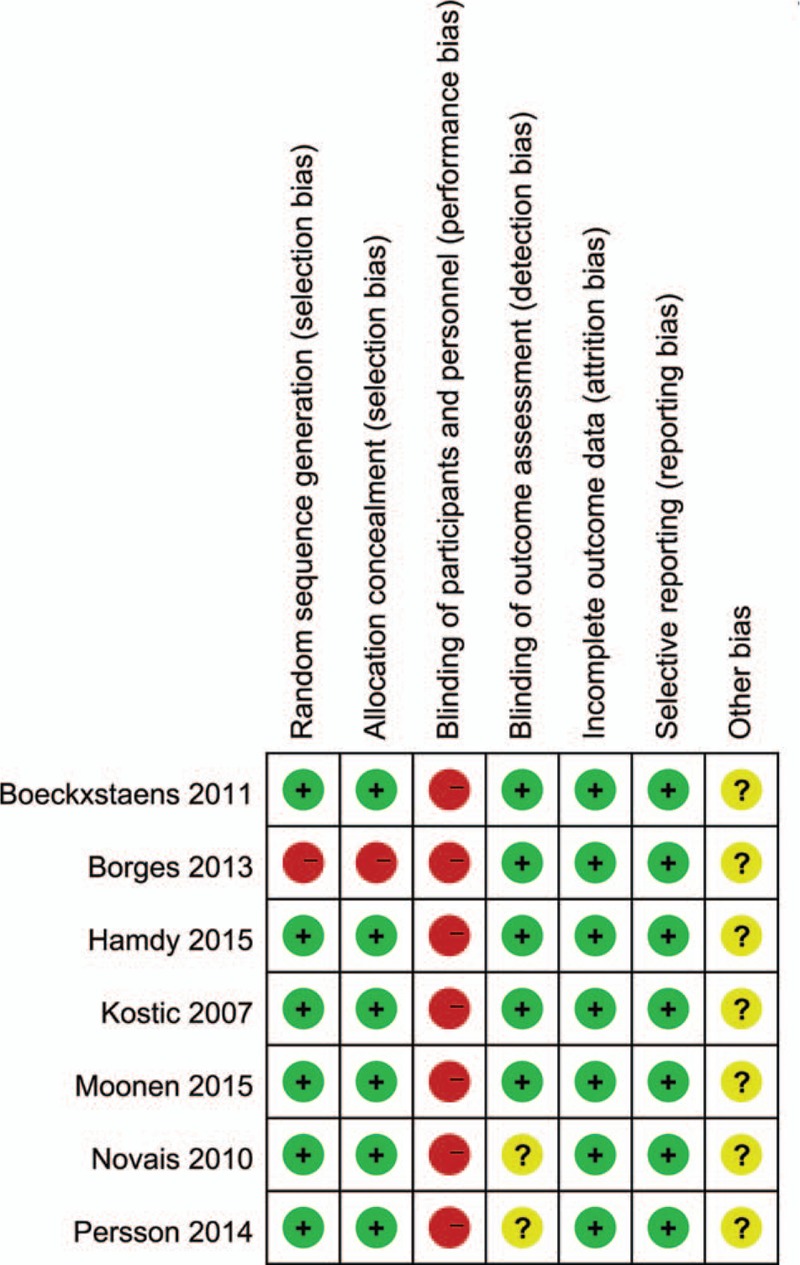
Risk of bias was assessed with use of the Cochrane risk-of-bias tool.

The quality assessment of trials is shown in Fig. 2. A funnel plot (Supplemental Content 2) showed that the studies were reasonably well scattered and did not suggest any publication bias.

### 3.3 Analysis of main outcome

#### 3.3.1 Remission at 3 months, and 1, 2 and 5 years

Data on remission rates following both treatments were available for 3 studies at 3 months,^[16–18]^ 4 studies at 1 year,^[10,14,16,17]^ 2 studies at 2 years,^[10,16]^ and 2 studies at 5 years.^[11,15]^

**Figure 3:**
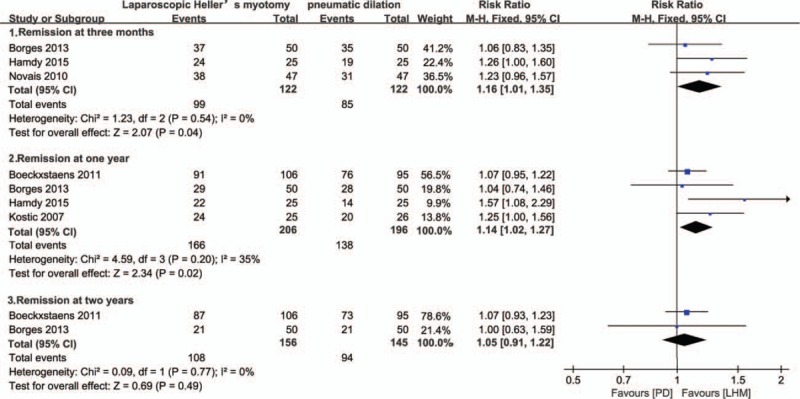
Remission rate at 3 months, and 1 and 2 years. CI = confidence interval, LHM = laparoscopic Heller myotomy, PD = pneumatic dilation.

**Figure 4:**
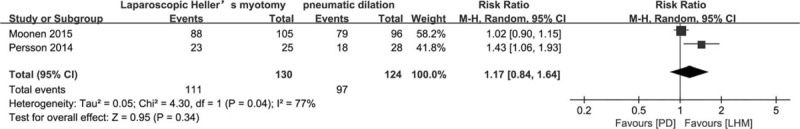
Remission rate at 5 years. CI = confidence interval, LHM = laparoscopic Heller myotomy, PD = pneumatic dilation.

At 3 months, 99 of 122 participants in the LHM group were in remission compared to 85 of 122 participants in the PD group, giving a risk ratio of 1.16 (95% CI 1.01–1.35, *P* = 0.04) (Fig. 3). At 1 year, 166 of 206 LHM participants were in remission compared to 138 of 196 PD participants, with a risk ratio of 1.14 (95% CI 1.02–1.27, *P* = 0.02) (Fig. 3). At 2 years, 108 of 156 LHM participants were in remission compared to 94 of 145 PD participants, with a risk ratio of 1.05 (95% CI 0.91–1.22, *P* = 0.49) (Fig. 3). At 5 years, 111 of 130 LHM participants were in remission compared to 97 of 124 PD participants, with a random-effects model risk ratio of 1.17 (95% CI 0.84–1.64, *P* = 0.34) (Fig. 4). Sensitivity analysis was performed by altering the statistical test (odds ratio or risk difference) and model (random-effects or fixed-effect) and did not change the results at the 3-month, and 1-year and 2-year analysis of remission. There was evidence of statistical heterogeneity in the 5-year remission analysis and random-effects model was applied.

### 3.4 Secondary outcomes

#### 3.4.1 Complications

**Figure 5:**
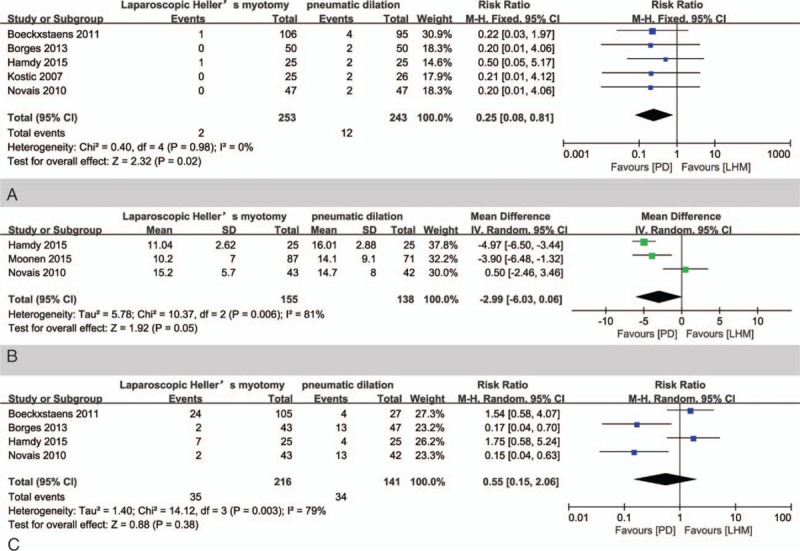
(A) Meta-analysis of adverse event rate to LHM versus PD in the treatment of achalasia. (B) Meta-analysis of posttreatment LOS pressure to LHM versus PD in the treatment of achalasia. (C) Meta-analysis of GER rate to LHM versus PD in the treatment of achalasia. CI = confidence interval, GER = gastroesophageal reflux, LHM = laparoscopic Heller myotomy, LOS = lower esophageal sphincter, PD = pneumatic dilation.

Boeckxstaens et al^[10]^ initially started PD with a 35-mm balloon and 4 perforations in 13 patients (30.8%) were found; the percentage was too high and a graded dilation approach was used, starting with 30-mm balloons. After excluding these 13 patients, the summary rate of adverse events requiring postoperative medical care in the fixed-effect meta-analysis from the 5 included studies was significantly lower with LHM than with PD, 2 of 253 LHM participants compared to 12 of 243 PD participants, with a risk ratio of 0.25 (95% CI 0.08–0.81, *P* = 0.02) (Fig. 5A).

#### 3.4.2 Posttreatment LOS pressure

Two trials did not report detailed data. Random-model meta-analysis of the other 3 trials showed that the mean LOS pressure after treatment was not significantly different in patients undergoing LHM versus those receiving PD with a mean difference of −2.99 (95% CI −6.03 to 0.66, *P* = 0.05) (Fig. 5B).

#### 3.4.3 Rate of GER

Four trials reported the rate of GER after treatment, defined as pH <4, >4.5% of the time in a 24-hour pH study.^[10,16–18]^ The rate of GER was not significantly different in patients undergoing LHM versus in those receiving PD with a mean difference of 0.55 (95% CI 0.15–2.06, *P* = 0.38) (Fig. 5C) by using random-model meta-analysis of these 4 studies.

#### 3.4.4 Improvement in quality of life

We could not perform a meta-analysis to compare the results of quality of life because only 2 trials measured it by using different instruments.^[10,14]^ There were no significant changes between quality of life of the patients treated with LHM and that of the patients treated with PD in the 2 trials, although both treatments improved the quality of life.

#### 3.4.5 Cost-effectiveness

Cost-effectiveness analysis was not performed in the present review as it was assessed only in 1 study.^[17]^ In the study, repeated sets of dilatation and the need for surgical treatment were considered during assessment of the cost of PD to avoid biased cost-effectiveness analysis; however, consideration of these conditions did not affect the significantly lower cost of PD (US$ 228) in comparison to that of LHM (US$ 580) (*P* = 0.0001).

## 4 Discussion

Three meta-analyses of RCTs comparing LHM with PD have been published^[6,8,9]^; however, in all of them, patient numbers were low and follow-up periods were short. Furthermore, all of them had flaws that might threaten the authenticity of their findings. A systematic review by Wang et al^[6]^ compared several therapeutic modalities used in patients with achalasia and included a small meta-analysis of LHM versus PD, in which 1 trial was not an RCT. Another meta-analysis by Yaghoobi et al^[9]^ compared 3-month remission rate with 1-year remission rate. The other systematic review by Schoenberg et al^[8]^ extracted 2 different styles for 1-year remission data: intention-to-treat analysis data extracted from 1 trial and per-protocol analysis data extracted from another trial; it is wrong to synthesize the 2 different styles of data together. After the 3 meta-analyses, several studies investigating LHM versus PD in the treatment of primary achalasia were published. The present study is the first attempt to perform a high-quality meta-analysis to compare the long-term efficacy, safety, and physiologic outcomes of LHM with PD in patients with newly diagnosed achalasia.

In the present meta-analysis, remission rates were greater at both 3 months and 1 year (short-term) for LHM compared to those for PD. There was no significant difference in remission rates within 2 and 5 years (long-term). However, due to incomplete data, of the 5 studies, 2 studies were excluded from the 3-month analysis, 1 study was excluded from the 1-year analysis, 3 studies were excluded from the 2-year analysis, and 3 studies were excluded from 5-year analysis. Short-term results are consistent with previous meta-analyses.^[8,9,19]^ Long-term results were first demonstrated by this study to have no significant difference. Other long-term data involve a cross-sectional study of a large cohort of achalasia patients treated at the Cleveland Clinic Foundation (the USA).^[20]^ This study clearly demonstrates a steady decrease in clinical efficacy for both graded PD and LHM to similar therapeutic success rates of 44% and 57% (not significant) at 6 years. As achalasia is a chronic disorder, the choice of treatment should be based on long-term rather than short-term results. This is especially of great clinical relevance as therapeutic success gradually decreases in time for both treatments and thus may lead to significant differences in outcome with a longer follow-up.

In the long-term remission rate analysis of 2 of 3 studies, the PD protocol allowing re-treatment of patients with recurrent symptoms has been criticized and forwarded as potential bias explaining the lack of superiority of LHM^[21]^; in fact, repeated dilation is internationally accepted and, most importantly, widely reflects daily clinical practice.^[22]^ Moonen et al report the exact repeat rate: after a median follow-up of >6 years, 25% of patients treated with PD required re-treatment, a figure comparable to previous studies.^[11]^

The most common complication after LHM or PD is perforation. In the present analysis, complication rates after LHM compared to PD were lower (0.8% vs 4.9%). Perforations that are managed intraoperatively without any consequences for the patient were not taken to be a complication in the present analysis. Boeckxstaens et al found a significantly higher complication rate (12%) and rated these perforations as complications.^[10]^ In the present study, procedure-related complications after PD were in the range of those found in previous studies.^[8]^

The postprocedural LOS pressure and the rate of GER were not significantly different between PD and LHM. There is 1 study that showed a correlation between the LOS pressure and clinical score (*r* = 0.29; *P* = 0.002) in 115 patients, although the correlation between severity of clinical symptoms and LOS pressure in achalasia remains controversial.^[23]^ Therefore, improvement in symptoms and the development of GER after treatment with PD or LHM may be partly explained by a decrease in LOS pressure.

Cost-effectiveness analysis was not performed in the present review as it was assessed only in 1 study. In the study, significantly lower cost of PD (US$ 228) in comparison to that of LHM (US$ 580) was found (*P* = 0.0001). Evaluating which treatment has lower cost needs more properly controlled data.

High-resolution manometry can classify subtypes of achalasia and can help to better direct treatment strategies for a more precise classification.^[24]^ Comparing different treatment strategies based on high-resolution manometry, no RCTs were found to date. Disease subtypes should be taken into account in future prospective studies to establish treatment recommendations, especially in light of new techniques such as peroral endoscopic myotomy, which has provided some promising preliminary results in a highly selected patient series treated in a few expert centers.^[25]^ There is a paucity of properly controlled data to assess this emerging technology at this time.

The weakness of our study is that both the number of studies and the number of participants randomized to either treatment were small. Future large, blinded RCTs with comparable treatment protocols and outcome assessment criteria are needed.

In summary, the results of the present review suggest that LHM compared with PD has a better short-term efficacy, but long-term remission rate has no difference. LHM has less immediate posttherapeutic adverse events. There were no significant difference in LOS pressure, GER rate, and quality of life. PD has more re-treatment.

## 5 Conclusions

There were no significant differences between LHM and PD in 2-year and 5-year remission rate. The present study indicates that either treatment can be proposed as the initial treatment for achalasia.

## Supplementary Material

Supplemental Digital Content
