# Supplementary material for: Laparoscopic Heller myotomy is not superior to pneumatic dilation in the management of primary achalasia: Conclusions of a systematic review and meta-analysis of randomized controlled trials
Source: Medicine (Baltimore). 2017 Feb 17;96(7):e5525. doi: 10.1097/MD.0000000000005525 (PMC5319488; doi:10.1097/MD.0000000000005525)

Supplemental Content 1: see Figure, Supplemental Content, which illustrates the Search Strategy


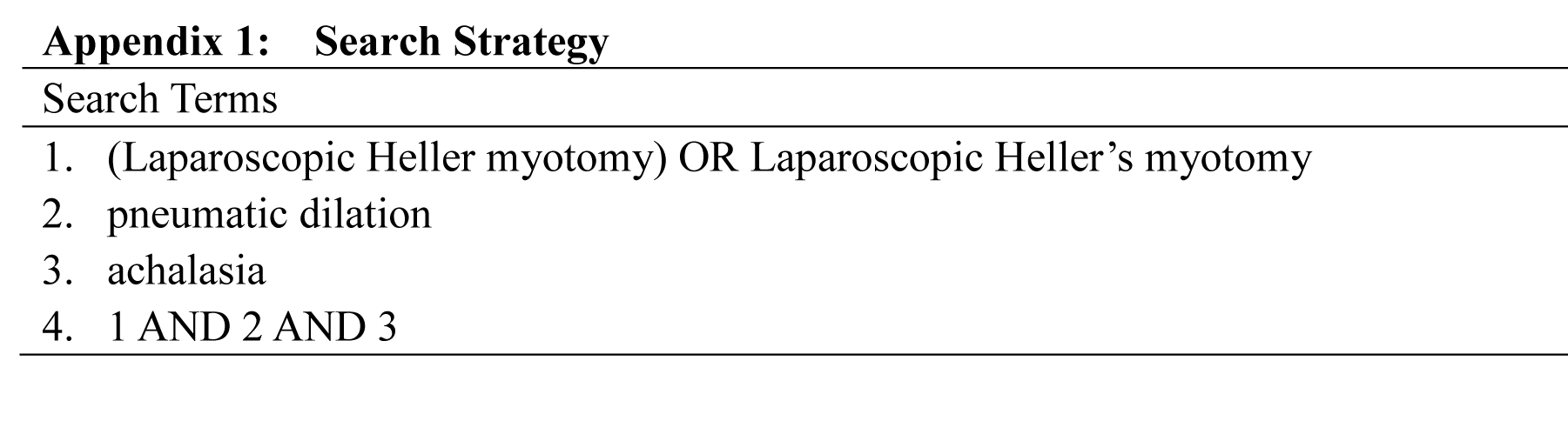


Supplemental Content 2: A funnel plot showed that the studies were reasonably well-scattered and did not suggest any publication bias.


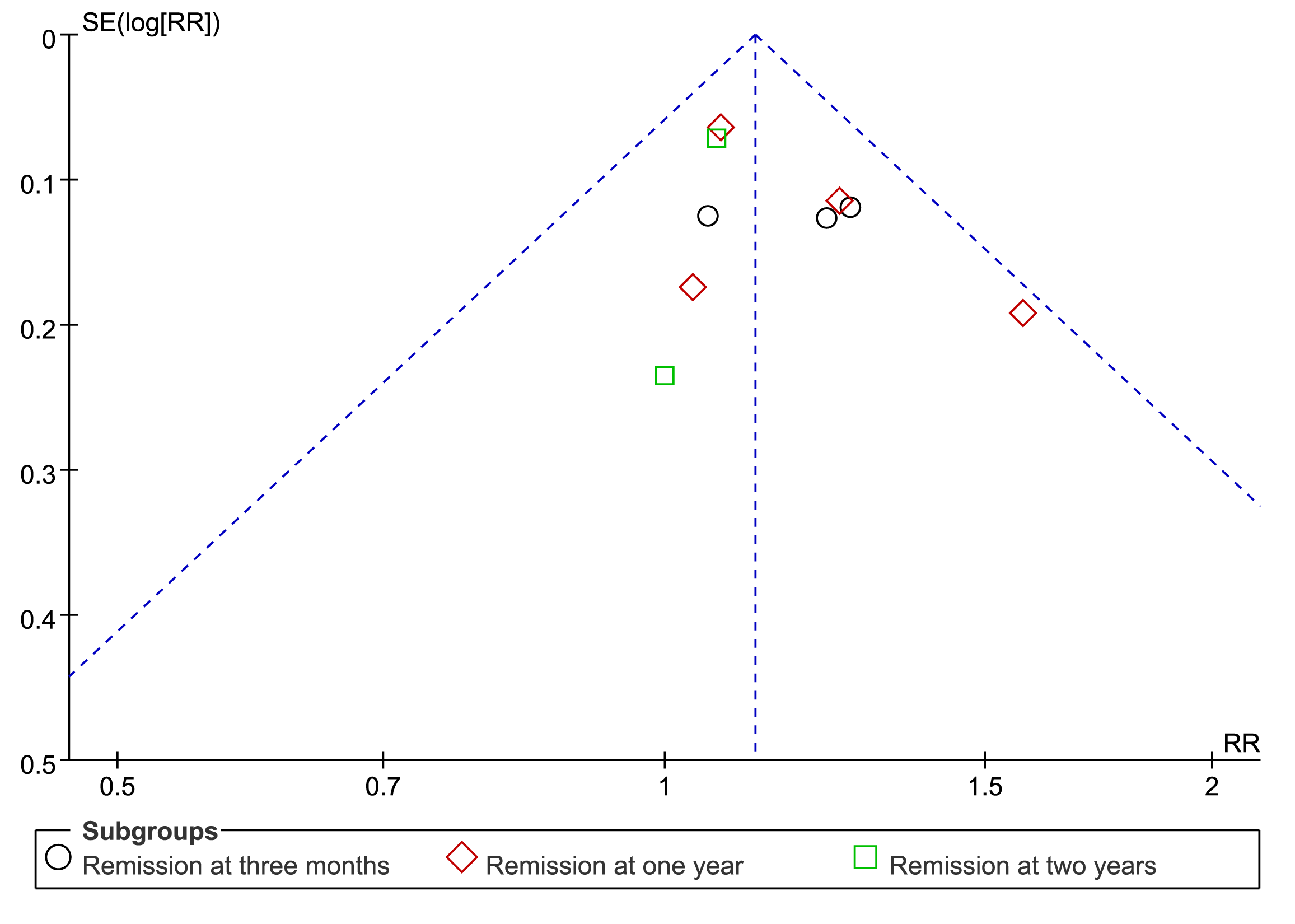

Supplement: Supplemental Digital Content [file medi-96-e5525-s001.doc]
